# Short-term treatment outcomes in human immunodeficiency virus type-1 and hepatitis B virus co-infections

**DOI:** 10.1186/s12941-016-0152-2

**Published:** 2016-06-02

**Authors:** Kwamena William Coleman Sagoe, Kwabena Obeng Duedu, Francesca Ziga, Afrakoma Adjoa Agyei, Theophilus Korku Adiku, Margaret Lartey, Julius Abraham Addo Mingle, Max Arens

**Affiliations:** Department of Medical Microbiology, School of Biomedical and Allied Health Sciences, University of Ghana, Korle-Bu, P. O. Box KB173, Accra, Ghana; Department of Biomedical Sciences, School of Basic and Biomedical Sciences, University of Health & Allied Sciences, Ho, Ghana; Pharmacy Department, Korle-Bu Teaching Hospital, Accra, Ghana; Department of Medicine and Therapeutics, School of Medicine and Dentistry, University of Ghana, Accra, Ghana; Retrovirus Laboratory, Department of Pediatrics, Washington University Medical School, St. Louis, MO USA

**Keywords:** Human immunodeficiency virus, Hepatitis B virus, Co-infection, Antiretroviral therapy, Drug resistance, Short-term therapy

## Abstract

**Background:**

Co-infection of HIV with HBV is common in West Africa but little information is available on the effects of HBV on short-term therapy for HIV patients. A 28 day longitudinal study was conducted to examine short-term antiretroviral therapy (ART) outcomes in HIV infected individuals with HBV co-infection.

**Methods:**

Plasma from 18 HIV infected individuals co-infected with HBV and matched controls with only HIV infection were obtained at initiation, and 7 and 28 days after ART. HIV-1 viral load changes were monitored. Clinical and demographic data were also obtained from patient folders, and HIV-1 drug resistance mutation and subtype analysis performed.

**Results:**

The presence of HBV co-infection did not significantly affect HIV-1 viral load changes within 7 or 28 days. The CD4^+^ counts on the other hand of patients significantly affected the magnitude of HIV-1 viral load decline after 7 days (ρ = −0.441, p = 0.040), while the pre-ART HIV-1 VL (ρ = 0.844, p = <0.001) and sex (U = 19.0, p = 0.020) also determined HIV-1 viral load outcomes after 28 days of ART. Even though the geometric sensitivity score of HIV-1 strains were influenced by the HIV-1 subtypes (U = 56.00; p = 0.036), it was not a confounder for ART outcomes.

**Conclusions:**

There may be the need to consider the confounder effects of sex, pre-ART CD4^+^, and pre-ART HIV-1 viral load in the discourse on HIV and HBV co-infection.

## Background

In West Africa, there is a high prevalence of hepatitis B virus (HBV) infection [1], and this is also reflected in similar high numbers of individuals with HIV also co-infected with HBV in the sub-region [2–7].

There are conflicting reports on the effects of HBV on the response to antiretroviral therapy (ART). Even though some studies have shown no significant effects of HBV infections on CD4^+^ recovery and/or suppression of HIV-1 plasma viral load (HIV-1 VL) during ART, [8–12] others have seen some effects [4, 5, 13–15]. Apart from having higher HIV-1 VL, individuals with HIV and HBV co-infection in Taiwan on ART were found to have more frequent virological failure compared to those with HIV alone [13]. In patients co-infected with HIV and HBV in Nigeria, the presence of baseline hepatitis B e antigen (HBeAg) was associated with a slower virological response (≤400 copies/ml) as compared to those without HBeAg at week 24 [4]. However, this effect of HBeAg did not reflect in viral suppression after 48 weeks of ART. CD4^+^ count recovery during ART has been shown to be initially blunted in individuals with co-infection as compared to those with HIV infection alone [15]. Also, large studies done in Africa and Europe have reported impaired CD4^+^ outcomes due to HBV co-infection after varying periods of ART [5, 14]. These seeming discrepancies in outcomes from the various studies may be attributed to different clinical factors. Since in vitro studies have shown that HBV protein X acts as a trans-activator for HIV infections [16, 17], there is the need to understand the effects of the interaction between HIV and HBV on short and long term ART outcomes, bearing in mind demographical, epidemiological, clinical and viral factors.

In the absence of long term HIV-1 VL data and challenges with adherence after the first few weeks of ART in developing countries, changes in HIV-1 VL after 7 days (HIV-1 VL_0–7_) and 28 days (HIV-1 VL_0–28_) of ART may be useful in predicting long-term virological outcome [18–23]. In a previous study we showed that the presence of HBeAg in HIV infected patients with HIV and HBV co-infection resulted in more severe immune suppression [7]. The objective of this study was therefore to use the same study population to determine the possible effects of co-infection with HBV on short-term ART outcomes in HIV infected patients.

## Methods

### Patients

Briefly, pre-ART plasma samples for a cohort of 138 patients were screened for HBsAg using a third generation ELISA, Surase B 96 (TMB), General Biologicals Corp, Hsin Chu, Taiwan, and the Core HBsAg rapid test, Core Diagnostics, Birmingham, UK. All positive samples were tested for hepatitis B e antigen and antibody (HBeAg and anti-HBe) using the EASE BN-96 (TMB) and IgM antibodies to hepatitis B core antigen (anti-HBc IgM) using the ANTICORASE MB-96 (TMB) kits respectively (General Biologicals, Taiwan). Controls for all HIV patients with HBV co-infection (n = 18) were chosen based on a baseline CD4^+^ > or < 100 cells/μl. HIV mono-infected patients were matched (1:1) to co-infected patients on CD4^+^ cell count. All patients were screened with a one-step anti-HIV 1&2 test (SD Bioline, Premier Medical Corporation Ltd., India) and were shown to be HIV-1 infected. Demographic and clinical information was obtained from patient hospital records and adherence measured by self-reporting and pill count. The various host factors examined as possible confounders in this study included age, sex, pre-ART CD4^+^, and WHO clinical disease staging, while the viral factors were HIV-1 subtypes and drug resistance mutations (HIV-1 DRMs).

### Ethics and consent

The study was approved by the University of Ghana Medical School Ethics and Protocol Review Committee and written informed consent was obtained from patients before enrolment in the study.

### HIV-1 RNA quantitation and determination of early decay

Aliquots of plasma frozen in volumes of 600 μl at −85 °C were used in HIV-1 VL determination using the COBAS Amplicor Monitor v1.5 tests and the COBAS Amplicor analyzer (Roche Diagnostics GmbH, Mannheim, Germany). To ensure that marginal differences did not affect viral load analysis, all plasma samples were frozen in aliquots to avoid multiple freeze-thaws, and new frozen plasma vials at −85 °C were mainly used in determining viral loads. HIV-1 VL was determined in duplicates at baseline, days 7 and 28 to reduce errors inherent within the Amplicor Monitor v1.5 assay. Since the lower limits of the standard monitor assay was 400 copies/ml, viral load values <400 copies/ml were considered as 400 copies/ml.

All HIV-1 VL analysis were done with the log values and plasma viral decay was measured as HIV-1 VL_0–7_ and HIV-1 VL_0–28_. Since HIV-1 VL change within 6 days after ART (HIV-1 VL_0–6_) <0.96 log or ≥1.68 log indicated a likelihood for poor and good response good response to ART respectively [21], the proportions of patients who had <0.96 log and ≥1.68 log were also determined. The proportion of patients with HIV-1 VL_0–28_ of ≥2.58 log which may reflect on the possibility of having sustained HIV-1 VL suppression was also done for patients with day 28 plasma samples [22].

### Primary HIV-1 mutations

Since HIV-1 DRM may be subtype related [24], the possible confounder role of primary HIV-1 DRMs and subtype on short-term ART was also investigated. The High Pure Viral RNA Kit (Roche Diagnostics GmbH, Mannheim, Germany) was used for the isolation of HIV-1 RNA in plasma. Partial polymerase genes (1065–1372 bp) were amplified with a nested PCR using six primers and a modified version of a protocol published previously [25]. Reverse transcription was done using the Titan One Tube RT-PCR system (Roche Diagnostics GmbH, Mannheim, Germany). The original protocol was modified to obtain polymerase (*pol*) gene fragment spanning all the protease (PR) and up to 230 amino acids in the reverse transcriptase gene (RT). The Techne TC-3000 thermocycler (Techne Incorporated, Burlington, NJ, USA), was used for all RT-PCR reactions. The RT-PCR products were cleaned with the High Pure PCR Product Purification Kit (Roche Diagnostics GmbH, Mannheim, Germany). The Big Dye Terminator Cycle sequencing kit v3.1 and the ABI PRISM 3100 Genetic Analyzer (Applied Biosystems International Incorporated, Foster City, USA) were used for forward and reverse sequencing. In addition, a third sequencing primer (RT-sec-1-S: 5′ CAA AAA TTG GGC CTG AAA ATC CAT A 3′) was used to ensure that the region towards the end of the nucleotides needed for the first 227 amino acids in the RT region were obtained. The ViroSeq HIV-1 Genotyping System v2 (Celera Diagnostics, Foster City, Calif, USA) was used as an alternative method to sequence the *pol* genes of HIV-1 strains which were difficult to amplify with the in-house assay [25].

Phylogenetic relationships were determined using a partial *pol* region containing 867 nucleotides and a RT region covering with 691 nucleotides and methods as described previously [26]. This was done to ensure that subtyping of the RT sequences was truly representative of the *pol* region. Sequences used for phylogeny and in the FASTA format were submitted to the Stanford University database (http://www.HIVdb.stanford.edu) for interpretation of resistance and assignment of subtype. Mutation scores were used to derive genotypic sensitivity scores (GSS) based on specific antiretroviral drugs used by patients, and this was done using comments from the Stanford database. If the mutation site was dimorphic then the total score was divided into two and the value used as the GSS. The GenBank accession numbers of the sequences used in this study are HQ170612–HQ170613, HQ170615, HQ170617–HQ170625, HQ529236–HQ529243, HQ529245, HQ529247, HQ529249, HQ529251, HQ529253–HQ529260, HQ529262 and HQ529263.

### Analysis of data

The various variables included age, gender, pre-ART CD4^+^, WHO clinical disease staging, HIV-1 subtypes, GSS, ARVs, patient’s adherence, pre-ART HIV-1 VL and changes within the first 28 days of ART, and co-infection status of patients. For analysis involving HIV-1 subtypes, the CFR02_AG strains and non-CRF02_AG were considered as separate groups.

The Mann–Whitney U and Kruskal–Wallis tests were used to compare the effects of HBV co-infection on HIV-1 VL_0–7_ and HIV-1 VL_0–28_. In order to assess the possibility of viral and host factors as confounders for HIV-1 VL_0–7_ and HIV-1 VL_0–28_ outcomes due to HBV co-infection, the Mann–Whitney U and Spearman’s correlation tests was used to determine the effects of the other study variables on HIV-1 VL_0–7_ and HIV-1 VL_0–28_ for all study subjects. Furthermore, since an HIV-1 VL decline <0.96 log or ≥1.68 log and ≥2.58 log may determine long term outcomes for patients on ART by day 7 and 28 respectively [18, 22], a stepwise bivariate logistic regression for HIV-1 VL decline <0.96 log or ≥1.68 log and ≥2.58 log was also done with all variables significantly affecting ART outcomes (pre-ART HIV-1 VL, pre-ART CD4^+^, and sex). All statistical analysis were done with SPSS Version 17 software (SPSS Inc., Chicago, Illinois) and the two-tailed p-values of <0.05 considered as significant.

## Results

A description of the demographic and clinical characteristics of HIV infected patients with HBV co-infection, and those with HIV infection alone is shown in Table 1. Generally, patients had low CD4^+^ counts with majority having WHO clinical stage 3.

**Table 1:** Description of study population

Variables^a^	Co-infected (n = 18)	HIV infections alone (n = 18)
Demographic data		
Sex, female (%, CI)	11 (61.1)	11 (61.1)
Age (median, IQR)	36.5 (33.8–41.5)	35.5 (30.8–45.2)
Clinical parameters		
CD4^+^ (median/IQR)	137 (35–197)	125 (50–182)
WHO disease stage		
2 (n = 3)	2 (11.1 %)	1 (5.6 %)
3 (n = 38)	15 (83.3 %)	13 (72.2 %)
4 (n = 5)	1 (5.6 %)	4 (22.2 %)
ARV combination		
d4T + 3TC + NVP (n = 8)	4 (22.2 %)	4 (22.2 %)
d4T + 3TC + EFV (n = 11)	4 (22.2 %)	3 (16.7 %)
CBV + NVP (n = 15)	5 (27.8 %)	6 (33.3 %)
CBV + EFV (n = 12)	5 (27.8 %)	5 (27.8 %)
Hepatitis virus markers		
HBeAg	10 (55.6 %)	–
Anti-HBe	8 (44.4 %)	–
Anti-HBc IgM	0 (0.0 %)	–

^a^For variable with n number of patients indicated, the percentages were calculated based on those values

### Polymerase gene polymorphisms

**Fig. 1:**
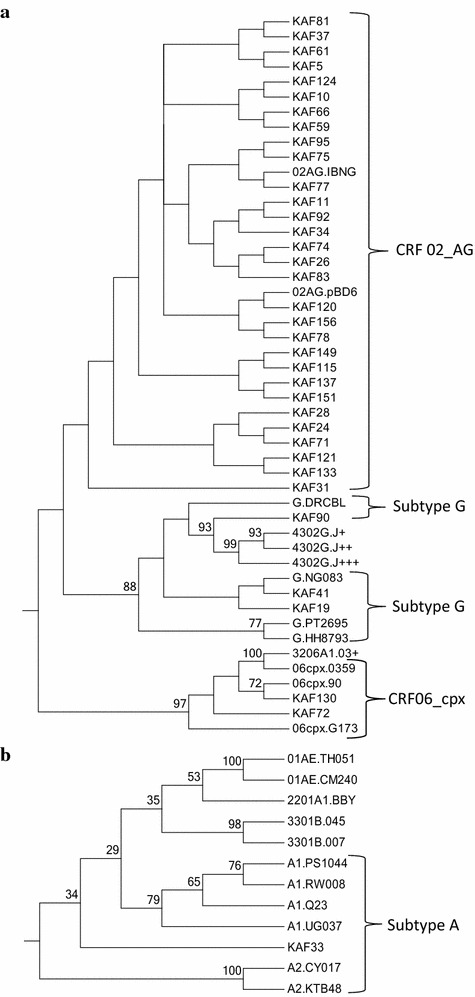
Phylogenetic trees using partial *pol* genes of HIV-1; **a** CRF02_AG, CRF06_cpx, and Subtype G clusters, and **b** Subtype A cluster. The reference subtype [accession number] include: **A1** (PS1044 [DQ676872]; Q23 [AF004885]; RW008 [AB253421]; UG037 [AB253429]), **A2** (KTB48 [AF286238]; CY017 [AF286237]), **B** (HXB2 [K03455]; 00T36 [AY423387]; BK132 [AY173951]; 1058 [AY331295]), **C** (BR025 [U52953]; ETH2220 [U46016]; IN21068 [AF067155]; SK164B1 [AY772699]), **D** (ELI [K03454]; 4412HAL [AY371157]; A280 [AY253311]; UG114 [U88824]), **F1** (VI850 [AF077336]; BR020 [AF005494]; FIN9363 [AF075703]; MP411 [AJ249238]), **F2** (0016BBY [AY371158]; MP255 [AJ249236]; MP257 [AJ249237]; 53657 [AF377956]), **G** (DRCBL [AF084936]; HH8793 [AF061641]; NG083 [U88826]; PT2695 [AY612637]), **H** (VI991 [AF190127]; VI997 [AF190128]; 056 [AF005496]), **J** (KTB147 [EF614151]; 7887 [AF082394]; 7022 [AF082395]), **K** (EQTB11C [AJ249235]; MP535 [AJ249239]), **01_AE** (CM240. [U54771]; TH051. [AB220944]), **02_AG** (pBD6 [AY271690]; IBNG. [L39106]), **06_cpx** (90 [AF064699]; 0359 [AY535659]; G173 [AB286851]), **09_cpx** (00IC [AJ866553]; 2911 [AY093605]), **22_01A1** (BBY [AY371159]), **28_BF** 12313 [DQ085872]; 12609 [DQ085873]; 12817 [DQ085874]), **29_BF** (16704 [DQ085876]; 11948 [DQ085871]), **32_06A1** (03+ [AY535660]), **33_01B** (007 [DQ366659]; 045 [DQ366662]), **34_01B** (2478 [EF165541]), **43_02G** (J+ [EU697904]; J++ [EU697907]; J+++ [EU697909]), **O** (ANT70 [L20587])

A total of 34 samples were amplified successfully using the two PCR methods and 30 were subtyped using phylogenetic analysis (Fig. 1). The relatively shorter sequences (KAF23, KAF56, KAF104 and KAF109) were subtyped solely using the Stanford database drug resistance program (http://www.HIVdb.stanford.edu). Majority of the subtypes were CRF02_AG, with 5 subtype G, 1 CRF06_cpx, 1 subtype A, and 1 CRF01_AE (Fig. 1), but the Stanford database drug resistance program failed to correctly infer subtypes A3 and CRF06_cpx (Table 2). The topologies of the 867 *pol* and 691 RT phylogenetic trees were similar thus confirming subtypes assigned to the RT region. There were no major drug resistance mutations but seven HIV-1 stains had some level of drug resistance (Table 2). The V108I mutation seen in KAF41, KAF71 and KAF104 reduces NVP and EFV susceptibilities by about twofold. The V118I mutation which is a polymorphic accessory NRTI-resistance mutation that occurs in combination with multiple TAMs, was seen in the only subtype A3 identified in the study (KAF33). Finally, the V179E mutation which causes low level resistance in NVP and EFV was seen in KAF69 and KAF71, but as a polymorphic site in KAF41 (V179EV) (Table 2). The GSS were influenced by the HIV-1 subtypes (U = 56.00; p = 0.036).

**Table 2:** HIV-1 subtype and drug resistance mutations in patients

Cases					Controls				
KAF	Subtype	Mutations^a^	GSS^a^	HIV-1 VL	KAF	Subtype	Mutations^a^	GSS^a^	HIV-1 VL
**151**	CRF02_AG	–	0	5.38	**075**	CRF02_AG	–	0	5.08
**083**	CRF02_AG	–	0	5.74	**124**	CRF02_AG	–	0	5.46
**092**	CRF02_AG	–	0	5.87	**023**	CRF01_AE	na	na	5.49
**028**	CRF02_AG	–	0	5.26	**130**	CRF02_AG/G	–	0	5.73
**090**	G	–	0	4.76	**033**	A/CRF01_AE	V118I	0	6.02
**056**	G	na	na	5.33	**081**	CRF02_AG	–	0	5.39
**071**	CRF02_AG	V108I, V179E	25	5.16	**078**	CRF02_AG	E138A	0	3.52
**120**	CRF02_AG	–	0	5.65	**104**	G	V108I	15	5.18
**026**	CRF02_AG	–	0	5.76	**149**	CRF02_AG	–	0	4.65
**115**	CRF02_AG	–	0	5.09	**074**	CRF02_AG	–	0	5.73
**031**	CRF02_AG	V90I	0	4.87	**137**	A/CRF02_AG	–	0	5.28
**061**	CRF02_AG	–	0	5.86	**010**	CRF02_AG	–	0	6.33
**077**	CRF02_AG	–	0	5.52	**064**	na	na	na	6.45
**041**	G	V108IV, E138A, V179EV	10	4.20	**121**	CRF02_AG	–	0	6.27
**156**	CRF02_AG	–	0	5.54	**037**	CRF02_AG	–	0	5.49
**011**	CRF02_AG	V90I	0	5.31	**095**	CRF02_AG	–	0	6.15
**109**	CRF02_AG	–	0	6.08	**019**	G	–	0	6.16
**200**	na	na	na	na	**024**	CRF02_AG	–	0	4.46

KAF, patients ID; Cases, individuals infected with HIV and HBV; Controls, those infected with only HIV; Mutations^a^/GSS^a^, HIV-1 drug resistance mutations/genotypic sensitivity scores (GSS) based on specific ARVs used by patients as determined by the Stanford database (analyzed on July 8, 2014); HIV-1 VL, pre-HAART HIV-1 plasma viral load. Subtypes were determined by the Stanford database

*na* not available

### Outcomes after short-term HAART

A total of 35, 22 and 20 HIV-1 VL quantitation results were available for pre-ART, day 7 and day 28 plasma samples respectively. The presence of HBV co-infection or any HBV marker did not significantly affect HIV-1 VL_0–7_ and HIV-1 VL_0–28_ (Table 3). Apart from sex, pre-ART CD4^+^, and pre-ART HIV-1 VL, all the other variables did not influence HIV-1 VL_0–7_ and HIV-1 VL_0–28_ (Table 4). The CD4^+^ of patients significantly affected the magnitude of HIV-1 VL_0–7_ (ρ = −0.441, p = 0.040), while the pre-ART HIV-1 VL also determined HIV-1 VL_0–28_ outcomes (ρ = 0.844, p = <0.001). HIV-1 VL decline <0.96 log or ≥1.68 log and ≥2.58 log was generally not affected by majority of the variables. Patients who had ≥1.68 log decline in HIV-1 VL were marginally likely to have higher CD4^+^ counts than those who did not achieve a 1.68 log decline (p = 0.065). The pre-ART HIV-1 VL was significantly higher in those who had achieved an HIV-1 VL decline of ≥2.58 log after 28 days of ART (p = 0.006).

**Table 3:** HIV-1 plasma viral loads in HIV infected patients with HBV infection

Groups	Mean rank	Mann–Whitney U	p value
Baseline			
HIV only (n = 18)	19.56	125.00	0.355
Co-infected (n = 17)	16.37		
Day 7			
HIV only (n = 11)	12.14	53.50	0.646
Co-infected (n = 11)	10.86		
Day 28			
HIV only (n = 10)	11.70	38.00	0.287
Co-infected (n = 10)	9.30		
VL_0–7_			
HIV only (n = 11)	10.00	44.00	0.287
Co-infected (n = 11)	13.00		
VL_0–28_			
HIV only (n = 10)	12.20	33.00	0.199
Co-infected (n = 10)	8.80

**Table 4:** Effect of host and HIV-1 viral factors on day 7 and 28 changes in HIV-1 plasma viral load

Variables	Categories	HIV-1 VL_0–7_ (N = 30)			HIV-1 VL_0–28_ (N = 22)		
		Mean rank	U/χ^2^ (df)	p value	Mean rank	U/χ^2^ (df)	p value
HBV status	HIV alone	10.00 (n = 11)	44.0	0.300	12.20 (n = 10)	33.0	0.218
	Co-infection	13.00 (n = 11)			8.80 (n = 10)		
Sex	Male	12.86 (n = 7)	43.0	0.503	15.35 (n = 9)	19.0	*0.020*
	Female	10.87 (n = 15)			8.29 (n = 11)		
WHO clinical stage	Stage 1	4.25 (n = 2)	2.794 (2)	0.247	9.00 (n = 1)	0.068 (2)	0.966
	Stage 2	12.09 (n = 17)			10.56 (n = 16)		
	Stage 3	13.00 (n = 3)			10.67 (n = 3)		
Antiretroviral drugs	d4T + 3TC + EFV	11.00 (n = 2)	0.737 (3)	0.864	10.50 (n = 2)	3.182 (3)	0.364
	d4T + 3TC + NVP	12.83 (n = 6)			9.75 (n = 4)		
	CBV + EFV	12.25 (n = 8)			13.13 (n = 8)		
	CBV + NVP	10.06 (n = 8)			7.50 (n = 6)		
Mutations	Present	5.75 (n = 2)	8.5	0.292	9.5 (n = 4)	26.0	0.878
	Absent	10.75 (n = 17)			10.75 (n = 16)		
HIV-1 subtype	02AG	10.41 (n = 17)	24	0.410	9.5 (n = 4)	37	0.898
	Non 02AG	13.50 (n = 4)			10.75 (n = 16)		

Significant p values are in italics

U, Mann–Whitney U test; χ^2^ (df), Kruskal–Wallis H test; Co-infection, HIV and HBV co-infection; *VL*_*0–7*_ HIV-1 plasma viral load changes after 7 days of therapy; *VL*_*0–28*_ HIV-1 plasma viral load changes after 28 days of therapy

A bivariate logistic regression using an HIV-1 VL decline <0.96 log revealed that CD4^+^ was marginally significant (p = 0.067). Also, pre-ART HIV-1 VL was marginally significant after a decline of ≥2.58 after 28 days of ART (p = 0.051).

## Discussion

Relatively few studies have evaluated the usefulness of early HIV-1 plasma viral decay, HIV-1 VL_0–7_ or HIV-1 VL_0–28_, in determining long term outcomes after initiation of ART or in determining the efficacy of ART regimens [18–22, 27–30]. One of such studies showed that HIV-1 VL_0–6_ was as good as using the decay constant in determining the likelihood for poor and good response to ART [21]. Furthermore, in a fairly large population of ART naïve adults, HIV-1 VL_0–7_ was significantly associated with HIV-1 VL after 48 weeks of ART but not after 24 weeks [18]. These studies therefore help to provide early warning indicators for long term outcomes of patients on ART. Even though a few patients in this study were estimated to have a reduced adherence this did not affect the short term outcomes of this study as has been seen elsewhere [22]. Thus with the relatively small population of individuals co-infected with HIV and HBV, adherence was not an issue in the analysis of the data. Admittedly, the lack of continuous HIV-1 VL data for all 36 patients may have created gaps in the analysis of data. In spite of this limitation, it is necessary to mention that the whole study population was considered in determining the effects of the different study variables on short-term ART outcomes.

In sub-Saharan Africa, individuals with HIV infections may report to ART clinics late and therefore not start ART when they are due [31]. This may reflect in low CD4^+^ counts which are indicators for treatment failure [32]. This is consistent with the parameters of the study population in which majority of the patients were classified as being in WHO clinical stage 3, and CD4^+^ affected outcomes after 7 days of ART. The HIV-1 VL_0–7_ has been shown to have a strong correlation with the phase 1 constant of HIV-1 decay [21, 23], and this correlation was weaker when calculated 3, 4 or 10 days after onset of ART but may be similar when done at day 6 [21]. The choice of day 7 HIV-1 VL as an indicator and the fact that CD4^+^ does affect its outcome, therefore suggests that those who are severely immunocompromised are more likely to have poorer outcomes during ART. The blunting of CD4^+^ in individuals with HIV and co-infected with HBV at different periods of ART [5, 33], may therefore affect HIV-1 VL outcomes at a point in time as shown by others [4]. This may partly have accounted for the observation that HBV co-infection may be associated with more frequent virological failure [13]. Pre-ART HIV-1 VL has been observed as one of the factors affecting long term outcomes in patients on ART [22]. Since this was also observed after 28 days on ART in this study, the use of outcomes after 7 and 28 days as representing long term outcomes to some extent is justified. Thus, in spite of the relatively small study population in this preliminary study, the results to a large extent are consistent with that which is observed in long term studies with specific respect to the effects of CD4^+^.

The studies cited earlier showing somewhat differences in ART outcomes were conducted in patients from different geographical regions. We postulated that since there is emerging data suggesting that HIV-1 subtype may influence the choice of antiretroviral drugs (ARVs) [24] then there is a possibility that the co-circulation of different subtypes may occur within different geographical areas. The presence of primary HIV-1 resistance which may be different in different subtypes may therefore serve as a confounder in looking at long term outcomes. Even though the GSS did not influence HIV-1 VL_0–7_ and HIV-1 VL_0–28_ significantly, the fact that those with HIV-1 subtype which were non CRF 02_AG strains had significantly higher GSS brings to the fore the hypothesis that regional subtype differences needs to be factored into the discussion of the effects of HBV on outcomes of ART. The possibility of primary HIV-1 DRMs influencing some virological outcomes in HBV co-infection in different geographical areas cannot be ruled out. This assertion is buttressed by the fact that primary HIV-1 DRMs are likely to result in treatment failure after 12 months of ART [34].

The hypothesis that HBV protein X may cause different outcomes in ART in those with and without HBV co-infection is not suggested in this study. However, CD4^+^, pre-ART HIV-1 VL and sex need to be considered as possible confounders. It is unclear how the latter will affect virological outcome.

As compared to other studies in Ghana, the mutations seen confirm the absence of major HIV-1 DRM in treatment naïve patients [35, 36]. Since no transmitted HIV-1 drug resistance mutations were seen, most of the mutations were more likely to be subtype or strain related. In spite of the small study population as compared to others [35, 37, 38], it seems that the V108I mutation may be fairly common in naïve patients.

## Conclusion

Pre-ART CD4^+^, pre-ART HIV-1 pVL, and sex may be important in determining ART outcomes in individuals on ART with HIV and HBV co-infections.
